# Comorbidity of Obsessive-Compulsive Disorder and Schizotypal Personality Disorder: Clinical Response and Treatment Resistance to Pharmacotherapy in a 3-Year Follow-Up Naturalistic Study

**DOI:** 10.3389/fpsyt.2019.00386

**Published:** 2019-06-17

**Authors:** Francesco Perris, Michele Fabrazzo, Valeria De Santis, Mario Luciano, Gaia Sampogna, Andrea Fiorillo, Francesco Catapano

**Affiliations:** Department of Psychiatry, University of Campania “Luigi Vanvitelli”, Naples, Italy

**Keywords:** obsessive-compulsive disorder, personality disorders, comorbidity, serotonin reuptake inhibitors, antipsychotics, augmentation strategy

## Abstract

The present study aims to analyze the clinical and socio-demographic characteristics of patients with obsessive-compulsive disorder (OCD) in comorbidity with schizotypal personality disorder (SPD), as well as the response rate to pharmacological treatments. OCD+SPD patients had a younger age at onset, a higher probability to have more severe obsessive-compulsive symptoms, a higher rate of schizophrenia spectrum disorders in their first-degree relatives, and a poorer insight compared to OCD patients. During the 3-year follow-up period, these patients showed a lower rate of recovery, thus requiring augmentation with different psychotropic medications, including low doses of antipsychotics. Our findings suggest that the comorbidity of OCD and SPD causes a poor treatment response, and a reduced probability to recover using standard pharmacological treatment strategies. Further investigations are needed to identify alternative strategies, including psychoeducation and cognitive behavioral therapy, to manage such frequent comorbidity in clinical practice.

## Introduction

Obsessive-compulsive disorder (OCD) includes a wide range of symptoms and is often associated with other psychiatric disorders (1). About 32–92% of OCD patients present at least one additional disorder, more often schizophrenia, major depression, or a personality or anxiety disorder, confirming that comorbidity in OCD is the rule rather than the exception (2, 3). The comorbidity with any other mental disorder increases the severity of OCD, worsens the prognosis, and influences the response to pharmacotherapy (4).

Despite not being the most frequent one, schizotypal personality disorder (SPD) has a clinical relevance among Axis II disorders when in comorbidity with OCD, with a 5–50% rate of co-occurrence reported in the different studies (5–9). This high range could be due to the different methodologies adopted in the various studies to evaluate the presence of SPD, such as the categorical vs. dimensional approach (10–13), the assessment instruments (structured clinical interviews, chart reviews), and the cohorts of patients (outpatients/inpatients, patients treated by specialistic clinics or community mental health centers) (14, 15). OCD patients with SPD (OCD+SPD) share several social and clinical characteristics, such as male gender, being unmarried, earlier onset of the disorder, higher prevalence of somatic and aggressive obsessions, and of counting and symmetry compulsions (5, 16). OCD+SPD patients frequently report a family history of learning difficulties, phobias and schizophrenia-related disorders, poor insight, worse social and work functioning, and psychotic-like experiences, such as delusional beliefs, suspiciousness, ideas of reference, magical thinking, and perceptual distortions (5, 6, 17–20). Moreover, several retrospective studies have found a worse prognosis and a poor response to SRI and cognitive behavioral therapy in OCD+SPD patients compared to patients without SPD (5, 7, 21–23). To our knowledge, no studies with long follow-ups have been conducted so far on the clinical response to pharmacological treatments commonly used to treat OCD in a sample of OCD+SPD patients. Therefore, we carried out a prospective, naturalistic study with the following aims: a) to identify the clinical and socio-demographic characteristics of OCD+SPD patients, and b) to assess their response to standard pharmacological treatments, compared to OCD subjects without SPD.

## Materials and Methods

### Subjects

Patients were recruited at the Department of Psychiatry of the University of Campania “L. Vanvitelli,” Naples, Italy. The study protocol has been submitted and approved by the Ethical Review Board of the University of Campania “L. Vanvitelli.”

Each participant gave his/her written informed consent upon full explanation of the study protocol. Patients were included in the study if they had the following inclusion criteria: 1) main diagnosis of OCD according to the DSM-IV and confirmed by the Structured Clinical Interview for DSM-IV Axis I Disorders (SCID-I) (24); 2) age between 18 and 65 years; 3) a minimum duration of illness of 1 year; and 4) willingness to participate in the study.

The exclusion criteria were as follows: 1) serious neurological illness, except tic disorder; 2) drug abuse or dependence; and 3) diagnosis of schizophrenia or other psychotic disorders.

### Baseline Assessment

All patients were assessed at baseline by the following instruments: 1) Structured Clinical Interview for DSM-IV Axis I Disorders (SCID-I) (24); 2) Structured Clinical Interview for DSM-IV Axis II Personality Disorders (SCID-II) (25); 3) Yale-Brown Obsessive-Compulsive Scale (Y-BOCS) (26); 4) Hamilton Rating Scale for Depression (HDRS), 17 items (27); and 5) Brown Assessment of Beliefs Scale (BABS) (28). The OCD symptoms were recorded using the Yale-Brown Obsessive-Compulsive Scale Symptom Checklist (Y-BOCS-SC), which includes 13 major categories of obsessions and compulsions. Patients’ socio-demographic (age, gender, marital status, years of education, employment) and clinical (age at onset, illness duration, type of onset, previous treatments for OCD, number of previous hospitalizations) characteristics were recorded on an *ad hoc* schedule.

The existence of any DSM-IV psychiatric disorder in first-degree relatives was analyzed using the Family History Research Diagnostic Criteria (29). Family history was recorded by direct interviews to patients and, whenever possible, to two close relatives.

The level of insight was assessed using the BABS, which is a seven-item rater-administered, semi-structured scale, designed to determine the degree of insight in various psychiatric disorders (28).

The ratings were made by clinicians with at least 5 years of experience in the management of OCD. Researchers were trained in the use of the assessment instruments by video- or audiotaped interviews, direct supervision, and calculation of the inter-rater reliability, which was very good for Y-BOCS, HDRS, BABS, and SCID diagnoses, with kappa values ranging from 0.75 to 0.90. All assessments were made by researchers not involved in the patient clinical management and who were not aware of the study aims.

### Treatment and Follow-Up

After the initial assessment, all patients were treated with a serotonin reuptake inhibitor (SRI). No difference was found regarding patients’ socio-demographic and clinical characteristics between patients who accepted the treatment program and those who refused it.

Since evidence suggests that SRIs are effective in the treatment of OCD, though with different profiles in terms of tolerability and side effects, medications were chosen on the basis of patients’ clinical characteristics, previous response to therapies, and psychiatrist’s clinical judgment.

SRIs were administered within recommended dosage ranges considered effective in OCD treatment; in particular, the following doses were used: 150–250 mg/day for clomipramine; 40–80 mg/day for fluoxetine; 150–300 mg/day for fluvoxamine; 40–80 mg/day for citalopram; 40–60 mg/day for paroxetine; and 100–225 mg/day for sertraline. Full-tolerated doses were maintained for at least 12 weeks. Response to treatment was defined as a decrease of at least 35% of the Y-BOCS total score from baseline. Patients who did not meet response criteria during the first drug trial underwent a flexible treatment, based on a sequential administration of different SRIs at maximum tolerated doses. During the follow-up, medications other than SRIs were used as therapeutic alternatives in treatment-resistant patients, according to the following dosage scheme: venlafaxine, 150–250 mg/day; mirtazapine, 30 mg/day; and imipramine, 150–250 mg/day. Patients who did not fully respond to SRIs received low-dose antipsychotics, such as pimozide, risperidone, and haloperidol. During the 3-year follow-up, patients were seen by their clinicians monthly during the first year and bimonthly thereafter; the frequency of visits varied according to patients’ needs. Patients’ clinical status was monitored by using Y-BOCS, HDRS, and BABS. Data about drug treatment (including dosage, side effects, and compliance) as well as hospital admissions were regularly recorded.

### Data Analysis

Descriptive statistics and percentages were used for demographic and clinical characteristics. Data have been analyzed using median, minimum and maximum values and nonparametric tests since a skewed distribution of continuous variables (e.g., age, mean score at HDRS, etc.) has been found. In particular, changes in Y-BOCS total and subtotal scores, HDRS, and BABS total scores during the follow-up period have been evaluated through Friedman test.

The Mann–Whitney test has been used for comparisons between OCD patients and OCD+SPD patients. During the follow-up period, patients were classified as in “partial” or “full” remission according to the score at Y-BOCS. In particular, “full remission” has been defined by a Y-BOCS total score below 8 for at least eight consecutive weeks, whereas “partial remission” by a Y-BOCS total score below 15 for at least eight consecutive weeks (30, 31).

At the end of the follow-up, patients have been grouped in “good outcome” and “poor outcome” according to the rate of partial remission. In particular, the “good outcome” group included patients reporting a partial remission rate higher than 40% of time-point assessments, while the “poor outcome” group included patients with a partial remission rate lower than 40% of time-point assessments (30, 31).

Statistical analyses were performed using the Statistical Package for Social Sciences (SPSS), version 17.0, and the level of statistical significance was set at p < .05.

## Results

### Global Sample and Attrition Rate

**Figure 1:**
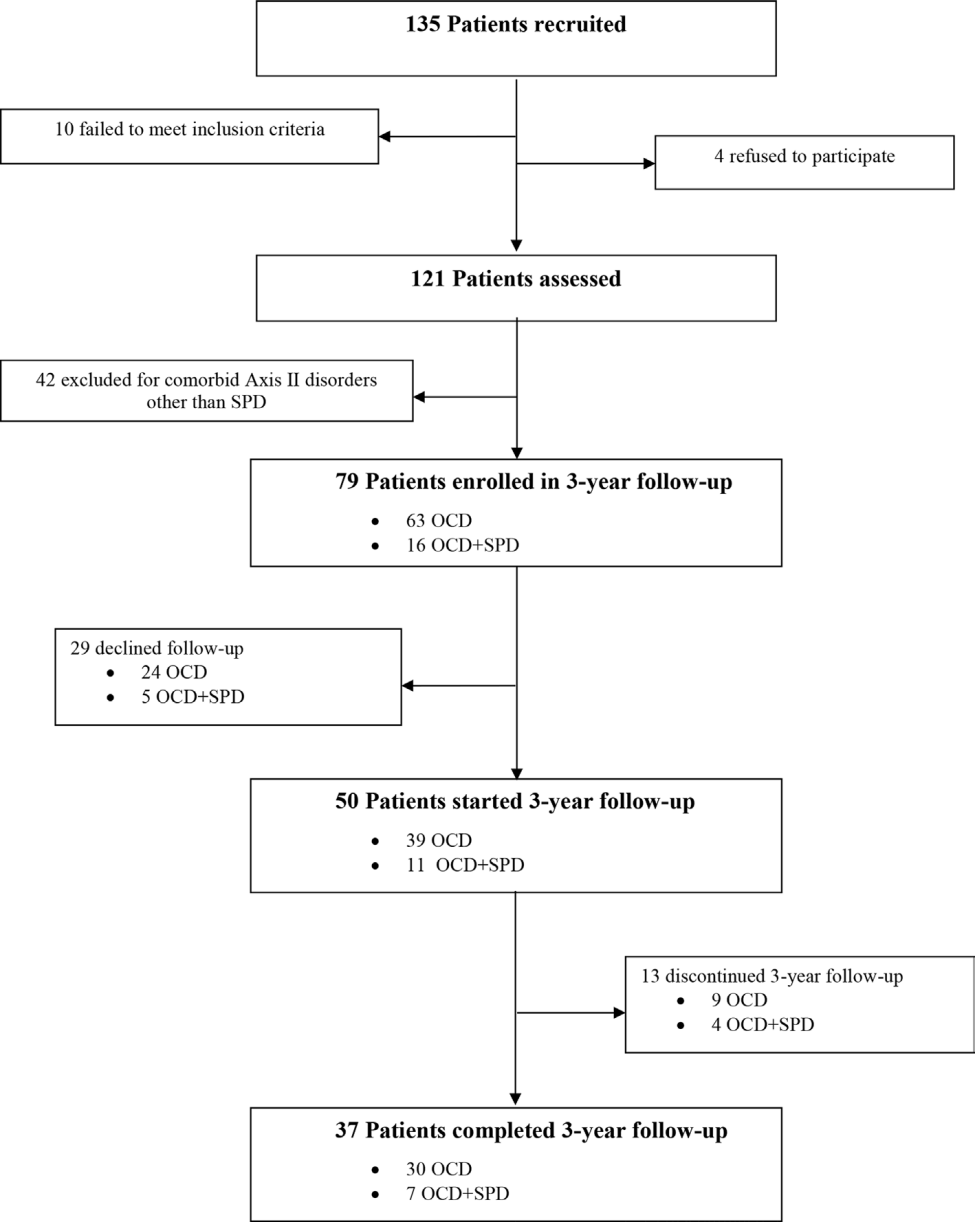
Flow chart of patients’ recruitment and attrition rate.

Attrition rate and reasons for exclusion are shown in **Figure 1**; 121 patients were assessed, and 42 patients were excluded due to the presence of comorbid mental disorders (anxiety disorders, N = 32; mood disorders, N = 24; tic disorders, N = 7; impulse control disorders, N = 16).

The final sample consists of 79 patients (40 male and 39 female), with a mean age of 31 (SD = 10.6; median: 30; min: 18; max: 60) years, and 10.8 (SD = 3.7; median: 12; min: 5; max: 19) years of education. About 50% of them were married and employed. The age at onset of OCD was 22.7 (SD = 9.5; median: 20; min: 12; max: 54) years, and the duration of illness was 8.3 (SD = 6.6; median: 7, min: 1; max: 33) years. A quarter of the sample (24%) was treatment-naive. Fourteen percent of patients (N = 11) had a family history of affective disorders, 10% (8 patients) of OCD, and 9% (7 patients) of schizophrenia-spectrum disorders. At baseline, the total Y-BOCS score was 26.4 (SD = 7.0); in particular, the mean scores for the obsessive-compulsive subscales for compulsion were 14.2 (SD = 2.9) and 12.2 (SD = 5.2) for obsession. The HDRS and BABS total scores were 14.6 (SD = 6.1) and 8.5 (SD = 4.9), respectively (**Table 1**).

**Table 1:** Socio-demographic characteristics of the sample.

	Global sample (N = 79)		OCD (N = 63)		OCD+SPD (N = 16)		*P-value*
	*M ± SD*	*m (min, max)*	*M ± SD*	*m (min, max)*	*M ± SD*	*m (min, max)*	
Age, years	31.0 ± 9.8	30 (18–60)	32.1 ± 10.2	31 (18–60)	26.7 ± 11.0	21 (18–54)	.018
Years of education	10.8 ± 3.8	12 (5–19)	10.7 ± 3.9	12 (5–19)	11.3 ± 2.3	11.5 (8–16)	.572
Age at onset	22.7 ± 8.6	20 (12–54)	24.1 ± 9.9	21 (12–54)	17.3 ± 5.0	16 (13–30)	.003
Duration of illness, years	8.3 ± 7.7	7 (1–33)	8.0 ± 6.6	6 (1–33)	9.4 ± 6.6	7.5 (2–24))	.372
Y-BOCS, total score	26.4 ± 6.8	27 (7–37)	25.6 ± 7.3	26 (7–37)	29.5 ± 4.8	31 (20–36)	.050
Y-BOCS, subscale compulsions	12.2 ± 4.9	13 (0–20)	11.5 ± 3.0	13 (0–20)	14.6 ± 2.5	15.5 (6–20)	.619
Y-BOCS, subscale obsessions	14.2 ± 3.0	15 (5–20)	14.0 ± 5.3	15 (5–20)	14.9 ± 3.7	14 (10–18)	.019
HDR-S, total score	14.6 ± 5.6	14 (4–34)	14.7 ± 6.4	14 (4–34)	14.3 ± 4.9	14 (6–25)	.888
BABS, total score	8.5 ± 4.6	7 (2–21)	7.4 ± 4.3	6 (2–19)	12.9 ± 4.9	14 (3–21)	.001
	**N (%)**		**N (%)**		**N (%)**		
Gender, male	40 (50.6)		29 (46)		11 (68.7)		.105
Marital status, married, yes	37 (46.8)		27 (42.8)		10 (62.5)		.160
Psychiatric disorders in the childhood, yes	20 (25.3)		10 (15.8)		10 (62.5)		.000
Employed, yes	37 (46.8)		32 (50.7)		10 (62.5)		.402
Family history of affective disorders, yes	11 (13.9)		9 (14.3)		2 (12.5)		.854
Family history of OCD, yes	8 (10.1)		7 (11.1)		1 (6.2)		.565
Family history of schizophrenia spectrum disorders, yes	7 (8.9)		2 (3.1)		5 (31.2)		.000

OCD, Obsessive-compulsive disorder; SPD, schizotypal personality disorder; Y-BOCS, Yale-Brown Obsessive Compulsive Scale; HDR-S, Hamilton Depression Rating Scale; BABS, Brown Assessment of Beliefs Scale; M, mean; SD, standard deviation; m, median; min, minimum; max, maximum.

The socio-demographic and clinical characteristics of OCD+SPD patients versus OCD patients are shown in **Table 1**. Patients with comorbid SPD had a younger age at onset, a higher likelihood of a family history of schizophrenia spectrum disorders, and of a personal history of psychiatric disorders in childhood. OCD+SPD patients report higher severity of OCD symptoms, as illustrated by Y-BOCS and Y-BOCS subscale for obsessions scores; a poorer insight, as resulted in a higher BABS total score. OCD+SPD patients report more frequent religious obsessions (31.3% vs. 9.5%, p < .05) and repetition compulsions (75% vs. 27%, p < .001) compared to OCD patients.

### Follow-Up Data

Twenty-nine (32.9%) out of the 79 patients refused to be followed up (OCD patients, N = 24; OCD+SPD patients, N = 5). No significant socio-demographic and clinical differences were found between patients who refused and those who accepted the follow-up assessments.

Among the 50/79 (63.3%) patients involved in the follow-up period, 13 (16.4%) dropped out during the 3-year period. In particular, 9/79 (9.4%) had a diagnosis of OCD without SPD; among these, eight dropped out in the first year and only one in the second year. Among patients with OCD+SPD, 2/79 (2.5%) dropped out in the first year, and the remaining two (2.5%) in the second year. Dropouts were mainly due to side effects (N = 7), lack of compliance to treatments (N = 5), and other reasons (N = 1). At baseline, there were no differences between patients who dropped out and those who completed the follow-up assessments as regards Y-BOCS total score, Y-BOCS obsessive subscale, Y-BOCS compulsive subscale, HDRS total score, and BABS total score. In summary, 42 out of 79 patients (53.2%) dropped out, specifically 9/79 (11.4%) in the OCD-SPD group, and 33/79 (41.8%) in the OCD without SPD group (**Figure 1**).

Among the 37 patients who completed the follow-up period, 10 patients (27%) were prescribed one SRI, 9 patients (24.3%) two SRIs, and 18 patients (48.6%) three or more SRIs. Eleven patients received low doses of antipsychotics as add-on treatment: risperidone (mean ± SD = 3.3 ± 1.1 mg/day, *n* = 4), haloperidol (2.6 ± 1.1 mg/day, *n* = 3), and pimozide (3.2 ± 1.1 mg/day, *n* = 4). When compared to OCD patients without SPD, OCD+SPD patients required different drug regimens (2.87 ± 2.61 vs 5.14 ± 1.67; *p* < .01), and were more likely to receive an augmentation with antipsychotics (7/30, 23.3% vs 5/7, 71.4%; *p* < .01). OCD symptoms improved in both groups, as shown by the reduction at the Y-BOCS total (OCD patients; p < .0001; OCD+SPD patients; p < .05) (**Table 2**). In particular, OCD patients over time reported a significant reduction of severity of both obsessions (p < .0001) and compulsions (p < .0001), while OCD+SPD did not show a reduction in the severity of compulsions (p = .103). Moreover, also depressive symptoms and insight gradually improved over time, as reported by the reduction of HDRS and BABS total scores (p < .000) (**Table 2**).

**Table 2:** Differences in clinical symptomatology during the 3-year follow-up period.

	**Baseline**		**3 months**		**12 months**		**24 months**		**36 months**		***p-value***
*** Y-BOCS total score***	***M ± SD***	***m (min, max)***	***M ± SD***	***m (min, max)***	***M ± SD***	***m (min, max)***	***M ± SD***	***m (min, max)***	***M ± SD***	***m (min, max)***	
**OCD**	25.6 ± 7.3	26 (7–37)	18.1 ± 9.1	19 (0–36)	14.8 ± 8.4	14 (0–30)	14.2 ± 8.2	14.5 (2–38)	13.6 ± 7.8	12 (2–34)	<.000
**OCD+SPD**	29.5 ± 4.0	31 (20–36)	28.4 ± 4.6	29 (20–37)	26.1 ± 5.7	29 (16–31)	25.1 ± 8.6	28 (0–34)	21.7 ± 11.8	25 (0–34)	<.018
*** Y-BOCS obsessions***											
**OCD**	14.0 ± 2.1	15 (5–20)	10.2 ± 4.2	10 (0–18)	8.2 ± 4.2	8 (0–17)	8.4 ± 3.9	8 (0–19)	7.6 ± 3.7	6 (2–15)	<.000
**OCD+SPD**	14.6 ± 3.6	14 (10–18)	13.3 ± 3.6	13 (10–19)	11.9 ± 2.6	12 (8–16)	11.4 ± 4.4	13 (0–18)	9.4 ± 5.7	10 (0–18)	<.007
*** Y-BOCS compulsions***											
**OCD**	11.5 ± 6.0	13 (0–20)	8.6 ± 5.4	9 (0–18)	6.9 ± 4.9	6 (0–16)	6.4 ± 5.1	6 (0–19)	6.1 ± 4.8	5 (0–19)	<.000
**OCD+SPD**	14.9 ± 2.4	15.5 (6–20)	15.1 ± 3.4	15 (8–20)	14.3 ± 4.3	14 (7–19)	13.7 ± 5.0	15 (0–19)	12.6 ± 6.8	15 (0–19)	<.103
*** HDRS***											
**OCD**	14.7 ± 4.3	14 (4–34)	8.7 ± 5.0	9 (0–18)	7.5 ± 4.9	7 (0–18)	7.8 ± 5.5	7 (0–32)	6.1 ± 3.8	6 (0–16)	<.000
**OCD+SPD**	14.3 ± 2.8	14 (6–25)	12.9 ± 3.1	12 (9–21)	12.9 ± 2.6	12 (6–15)	11.0 ± 2.5	10 (0–15)	9.1 ± 4.7	9 (0–14)	<.000
*** BABS***											
**OCD**	7.6 ± 4.7	6 (2–19)	6.4 ± 4.3	4 (2–18)	5.6 ± 3.8	4 (2–18)	5.3 ± 3.4	4 (3–16)	5.3 ± 3.1	4 (3–15)	<.000
**OCD+SPD**	12.9 ± 4.9	14 (3–21)	14.1 ± 4.7	14 (6–19)	12.3 ± 4.7	13 (6–18)	12.3 ± 4.4	15 (5–17)	12.1 ± 4.7	14.5 (5–16)	<.000
*OCD, Obsessive-compulsive disorder; SPD, schizotypal personality disorder; Y-BOCS, Yale-Brown Obsessive Compulsive Scale; HDR-S, Hamilton Depression Rating Scale; BABS, Brown Assessment of Beliefs Scale; M, mean; SD, standard deviation; m, median; min, minimum; max, maximum.*											

At the end of the follow-up, 33.3% (*n =* 10) of OCD patients and 85.7% (*n* = 6) of OCD+SPD subjects still met OCD criteria. Nine patients were in “full remission” at the end of the follow-up (eight from the former group and one from the OCD-SPD group). Twelve (40%) patients from the pure OCD group were in partial remission, compared with no one from the OCD+SPD group (**Table 3**). On the other hand, a “good outcome” was reached only by 60% (18 out of 30) of OCD patients compared with no one from the OCD+SPD group (**Table 3**).

**Table 3:** Final outcome after 3-year follow-up in OCD and OCD+SPD patients.

	OCD (*n* = 30)	OCD+SPD (*n* = 7)	*p*
**Full remission, n (%)**	8 (26.7)	1 (14.3)	NS
**Partial remission, n (%)**	12 (40)	0 (0)	<.042
**No remission, n (%)**	10 (33.3)	6 (85.7)	<.012
**Good outcome, n (%)**	18 (60)	0 (0)	<.004

“Full remission”: Y-BOCS total score below 8 for at least eight consecutive weeks.

“Partial remission”: Y-BOCS total score below 15 for at least 8 weeks.

“Good outcome”: partial remission for more than 40% of the follow-up period.

NS: not significant; OCD, Obsessive-Compulsive Disorder; SPD, schizotypal personality disorder.

## Discussion

To our knowledge, this is one of the longest prospective and naturalistic follow-up studies on the outcome of OCD+SPD patients. Our findings show that this subgroup of patients is resistant to routine pharmacological agents and has a poorer outcome compared to pure OCD patients.

Our main finding is that OCD+SPD patients probably represent a distinct subgroup of OCD patients who have a low probability of symptom remission after standard pharmacological treatments (7, 21, 22). In fact, excluding one patient with a full remission, most of the patients fulfilled only partial remission criteria during a period longer than 40% of the 3-year follow-up. These patients have an early onset of the disease, a family history of schizophrenia-spectrum disorders, a history of childhood psychiatric disorders, a higher illness severity, and lower levels of insight. This finding confirms what was found by Dell’Osso et al. (32), who reported that early onset of OCD is associated with a more severe and disabling course of the illness, treatment resistance, and worse personal and work functioning.

Nevertheless, OCD+SPD patients showed a reduction of obsessive-compulsive and depressive symptoms, as well as an improvement in the level of insight, in coping strategies with their disease, and a better quality of life. Therefore, our results emphasize the need to develop individualized treatment strategies in order to obtain a complete symptom remission in this subgroup of patients (33). Several guidelines (34, 35) for the pharmacological treatment of resistant OCD patients suggest to augment the pharmacological therapy using low doses of antipsychotic medications. According to these guidelines, in our clinical sample, five patients with OCD+SPD received this kind of augmentation strategy. The efficacy of this pharmacological strategy, compared to standard SRI treatment, should be more widely investigated as it might represent a routine therapeutic protocol, or even a first-line treatment for these patients (36).

Most studies (7, 21, 22) have found that the outcome of patients with OCD is worse when there is a SPD in comorbidity. Poyurovsky et al. (6) suggest that schizotypal traits may be influenced by a genetic predisposition, and confirm the higher prevalence of schizophrenia-spectrum disorders according to this criterion, which might be applied to make OCD+SPD diagnosis.

Our data confirm that the levels of insight is reduced in OCD+SPD patients, as shown by a decrease in the BABS total score during the 3-year follow-up (21).

The severity of OCD is higher in OCD+SPD patients with predominantly sexual and religious obsessions along with repetitive rituals. This finding is contrary to that of Tallis and Shafran (16), who found higher levels of checking and ordering obsessions, and counting compulsions in this group of patients. Sobin et al. (5) found a predominance of counting and ordering compulsions, along with prevalent aggressive and somatic obsessions. Comparing our findings with previous studies, we can conclude that patients with OCD+SPD do not have a specific symptomatological pattern, but the occurrence of symptoms is highly dependent from context.

These data confirm the existence of a specific nosographic entity laying between OCD and schizophrenia, called schizo-obsessive spectrum disorder. This syndrome has clear and well-defined clinical, phenomenological, genetic, neurobiological, and neurocognitive characteristics (37). This spectrum includes OCD, OCD with poor insight, OCD with SPD, schizophrenia with obsessive-compulsive symptoms, schizophrenia with OCD, and pure schizophrenia (6).

The main limitation of our study is the small sample size, due to a high dropout rate of more than 50% of patients interviewed at baseline. However, our center—being highly specialized in the treatment of OCD—takes in charge patients from a wide geographical area. Therefore, the difficulties in reaching the center may represent an obstacle for patients to attend all visits during the follow-up period. Moreover, our patients did not receive any reimbursement for their follow-up visits. It may be that providing patients with financial support, or with home visits, may prevent them from dropping out, due to costs of transportation and working days loss in long-term longitudinal studies.

Another relevant limitation of our study is represented by the limited use of cognitive behavioral therapy (CBT), which is known to be a first choice treatment for patients with resistant OCD, as happens in routine practice (38–41). However, this was due to the fact that the primary focus of our study was on long-term pharmacological treatment of OCD with and without SPD, and therefore we did not primarily focus on the use of CBT and other psychotherapies as add-on treatments. The use of CBT on a large scale requires further investigation to explore its wide potentiality and attest its efficacy. Although experts consider CBT as a viable alternative in case of SRI resistance, few data are available on its use in partial or non-responder patients to medications (38). Another limitation is due to the adoption of the DSM-IV criteria for patients’ recruitment, which could limit the generalizability of results. This methodological choice is due to the longitudinal nature of our study, which started before the introduction of the DSM-5. Therefore, we aim to improve the sample size of our study, adopting the DSM-5 criteria in order to evaluate the stability of our findings.

Finally, we must acknowledge that the categorical approach to schizotypy was adopted through the use of SCID-II. Since this choice could have reduced the identification of some potentially significant effects and we aim to carry on further studies using a dimensional measure of schizotypy, such as the Schizotypal Personality Questionnaire (42).

## Conclusions

Patients with OCD+SPD showed a worse response to treatments and were less likely to recover from OCD, compared to OCD patients without SPD. Our findings suggest that OCD+SPD might represent a clinical entity *per se*, which is different from pure OCD, and this may be the reason why it does not fully respond to conventional anti-obsessive treatments. However, it would be helpful to identify the neurobiological correlates of OCD+SPD comorbidity. Larger studies with longer follow-ups are needed to establish the benefits of an augmentation strategy with low doses of antipsychotics or psychosocial interventions, such as family psychoeducation or CBT, considering the frequent family history of schizophrenia-spectrum disorders and the low levels of insight in these patients (43–45). Also, modern brain stimulation techniques may result to be effective in this subgroup of patients.

## Ethics Statement

The study has been approved by the Ethical Review Board of the University of Campania “Luigi Vanvitelli.”

## Author Contributions

FP, MF, VDS, ML, FC, and AF designed the study and wrote the first draft of the paper. GS and VDS managed the literature search and the statistical analysis. FP, AF, and MF developed the second and final version of the paper.

## Conflict of Interest Statement

The authors declare that the research was conducted in the absence of any commercial or financial relationships that could be construed as a potential conflict of interest.
